# Key factors predicting suspected severe malaria case management and health outcomes: an operational study in the Democratic Republic of the Congo

**DOI:** 10.1186/s12936-022-04296-2

**Published:** 2022-09-27

**Authors:** Jean Okitawutshu, Aita Signorell, Jean-Claude Kalenga, Eric Mukomena, Giulia Delvento, Christian Burri, Fatou Mwaluke, Valentina Buj, Moulaye Sangare, Sylvie Luketa, Nina Brunner, Tristan Lee, Manuel Hetzel, Christian Lengeler, Antoinette Tshefu

**Affiliations:** 1 Swiss Tropical and Public Health Institute, Allschwil, Switzerland; 2 University of Basel, Basel, Switzerland; 3 Kinshasa School of Public Health, Kinshasa, Democratic Republic of the Congo; 4 School of Medicine, Department of Public Health, University of Lubumbashi, Lubumbashi, Democratic Republic of the Congo; 5 Clinton Health Access Initiative, Kinshasa, Democratic Republic of the Congo; 6 UNICEF, New York, NY USA; 7 UNICEF, Kinshasa, Democratic Republic of the Congo

**Keywords:** Democratic Republic of the Congo, iCCM, IMCI, Severe malaria, Rectal artesunate, Injectable artesunate

## Abstract

**Background:**

Evidence suggests that pre-referral Rectal Artesunate (RAS) can be a life-saving intervention for severe malaria in remote settings in Africa. Recognition of danger signs indicative of severe malaria is critical for prompt and appropriate case management.

**Methods:**

This was an observational study conducted in three Health Zones of the Democratic Republic of the Congo to determine the distribution of dangers signs for severe malaria and assess their impact on RAS use, referral completion, injectable treatment and ACT provision, and health outcomes including death. An individual-level analysis was carried out, using multilevel-mixed effects logistic regression models. Severely ill febrile children < 5 years seeking care from community-based healthcare providers were recruited into a patient surveillance system based on the presence of key danger signs. Clinical and case management data were collected comprehensively over a 28 days period. Treatment seeking was elicited and health outcomes assessed during 28 days home visits.

**Results:**

Overall, 66.4% of patients had iCCM general danger signs. Age of 2–5 years and iCCM general danger signs predicted RAS use (aOR = 2.77, 95% CI 2.04–3.77). RAS administration positively affected referral completion (aOR = 0.63, 95% CI 0.44–0.92). After RAS rollout, 161 children died (case fatality ratio: 7.1%, 95% CI 6.1–8.2). RAS improved the health status of the children on Day 28 (aOR = 0.64, 95% CI 0.45–0.92) and there was a non-significant trend that mortality was higher in children not receiving RAS (aOR = 1.50, 95% CI 0.86–2.60). Full severe malaria treatment at the RHF including injectable anti-malarial and a course of ACT was highly protective against death (aOR = 0.26, 95% CI 0.09–0.79).

**Conclusions:**

The main findings point towards the fact that danger signs are reasonably well recognized by health provider at the primary care level, and that RAS could influence positively health outcomes of such severe disease episodes and death. Its effectiveness is hampered by the insufficient quality of care at RHF, especially the provision of a full course of ACT following parenteral treatment. These are simple but important findings that requires urgent action by the health system planners and implementers.

**Supplementary Information:**

The online version contains supplementary material available at 10.1186/s12936-022-04296-2.

## Background

In 2020, an estimated 241 million cases and 627,000 deaths due to malaria occurred worldwide, of which 228 million (95%) and 602,000 (96%) were in Africa [1]. If not appropriately treated, severe malaria (SM) often leads to death or irreversible sequelae [2–5]. Prompt, effective anti-malarial treatment coupled with quality supportive care can substantially reduce severe malaria mortality rates [6, 7], although a high average case fatality rate (CFR) of 8.7% was found in a high-quality multi-centre trial in Africa [8]. One of the major challenges remains the limited access to higher-level health facilities, especially for populations living in remote areas, resulting in treatment delays of several hours or even days [9, 10]. Injectable artesunate (AS) is the recommended first line treatment of severe malaria as compared to parenteral quinine [8, 11–14]. When delays in reaching referral health facilities (RHF) are expected, the World Health Organization (WHO) recommends pre-referral treatment, either with a single dose of a parenteral anti-malarial, or with a single dose of rectal artesunate (RAS) [6]. RAS is also recommended as a pre-referral treatment in the integrated community case management (iCCM) guidelines [15, 16] and for primary health care facilities (PHC) where injectable anti-malarials are often not available [6, 17, 18]. In clinical settings, RAS was shown to be an excellent anti-malarial, fast acting, and safe and well accepted [19–21]. Its efficacy in reducing child mortality was shown in a large randomized placebo-controlled clinical trial in Bangladesh, Tanzania and Ghana [22]. However, its potential effectiveness as a life-saving intervention under real-world conditions remains to be demonstrated [23]. The DRC has the second highest malaria mortality burden worldwide, with high average prevalence rates [24–28] almost everywhere and at least 45,000 deaths per year [1, 29]. It has a high CFR for hospitalized malaria (28%) in some settings [30], particularly in the many hard-to-reach areas of this massive country. Although the country has markedly improved both the prevention and case management of malaria in the recent decade [17, 31], including the implementation of iCCM packages, new interventions are urgently required to address the high number of childhood deaths resulting from malaria. In order to achieve this, much remains to be done in better understanding the burden and patterns of severe febrile illnesses at community level, treatment seeking and its determinants, as well as the circumstance of deaths from malaria. Obviously, better management of severely ill children, who are at a high risk of dying, is of high priority to reduce the unacceptably high mortality in Congolese children. In some settings, the CFR for hospitalized severe malaria can be as high as 28% [30], which is well above the < 10% in high quality care settings [8].

The results presented here are part of the Community Access to Rectal Artesunate for Malaria (CARAMAL) project carried out in the DR Congo, Nigeria and Uganda to assess the case management for SM in remote locations and assess the public health value of RAS as a pre-referral treatment under real-world conditions [32]. The design and main impact results for the three sites are presented elsewhere [32, 33].

The aim of the present work was to describe for the DRC the distribution of severity signs and symptoms, among children < 5 years with regard to an episode of severe febrile illness/suspected SM. In a second step, the predictive value of danger signs and symptoms on a number of main study outcomes was assessed: likelihood of RAS use, referral completion, administration of injectable artesunate in a Referral Health Facility (RHF), and health outcomes including clinical cure and mortality.

## Methods

### Study site

This study was conducted in three rural Health Zones (HZ) in western DRC: Kenge in Kwango Province, Ipamu and Kingandu in Kwilu Province (Additional file 1: Fig. S1), with an estimated population of 786,000 inhabitants, of which 145,000 children < 5 years (https://www.worldpop.org, 2018). The selection of the study areas was driven firstly by operational considerations, such as having a functioning iCCM programme supported by UNICEF, secondly by a presumably functioning referral system, and finally it had to be in an area of acceptable security. Then a sufficient population to reach the sample size calculated from an assumed baseline CFR of 6% and the ability to detect a 30% decrease in CFR following RAS roll-out was selected [32].

In the selected areas, the peripheral care system was composed of 42 functioning Community Health Care Sites (CHCS) and 152 Primary Health Care facilities (PHC) from the public, missionary and private sectors. The reference care level comprised 19 RHF including 16 Referral Health Centers and 3 General Referral Hospitals.

CHW are trained on iCCM algorithms, while nurses at PHCs follow the Integrated Management of Childhood Illness (IMCI) strategy. Both cadres provide a minimum package of preventive and curative care including RAS provision and referral of severe cases. By contrast, RHFs are staffed by medical doctors and offer a much more comprehensive package of care, including blood transfusions and the management of clinical complications. Distances between CHWs and their nearest RHF were often large, with a median of distance = 17 km (9–22), leading to an estimated median referral time of 2.75 h (2.0–3.25). There was no organized public transportation system, so patients mainly moved by foot or bicycle.

### Study design

CARAMAL was an observational study based on a before-and-after plausibility design [34] in the framework of the RAS roll-out through established CHCS and PHC. The core of the study evaluation was a Patient Surveillance System (PSS) maintained over the two study phases: (1) pre-RAS for 10 months before RAS rollout (from June 2018 to March 2019) and (2) post-RAS that lasted 16 months after RAS introduction (from April 2019 to July 2020). The PSS allowed to enroll eligible children since the first point of contact with the health care system and track them comprehensively up to Day 28. Health care providers at all levels, including CHWs, PHCs and RHFs, underwent training sessions on the effective use of RAS according to the country’s iCCM and IMCI guidelines. An extensive description of the study design, sites and methods is available elsewhere [32].

### Definition of relevant danger signs

- *iCCM general danger signs* These consisted of the general danger signs according to the iCCM algorithm and included: (1) vomiting everything, (2) convulsions, (3) not being able to drink/eat, and (4) being very sleepy or even unconscious [16]. The presence of at least one of these danger signs triggered RAS administration and immediate referral in children under 6 years old at community level [6, 18].
- *DRC-specific iCCM danger signs* Two additional signs/symptoms in wide use in the DRC identifying a child as being eligible for referral and hence RAS pre-referral treatment: (1) being “unable to sit or stand up” and (2) “weakness or asthenia” were also considered (Additional file 2: Fig. S2 and Table 2).

### Participants

All children who were seeking care at a CHW or PHC setting that fulfilled the following inclusion criteria were enrolled: (1) children under 5 years of age, (2) fever or a history of recent fever, (3) presence of at least one of the “*iCCM general danger signs” or “DRC-specific iCCM danger signs”*, and (4) provision of signed consent by parent /guardian. Those aged more than 5 years old or without permanent resident in study area were excluded.

### Procedures

#### Enrolment

A child fulfilling the inclusion criteria was provisionally enrolled into the PSS by a trained CHW or PHC nurse following its first contact with the health system. After a clinical assessment and a positive malaria rapid diagnostic test (mRDT) the child was considered as a suspected case of SM, given RAS and referred to a designated RHF. Information such as address, child’ and parent’s demographics as well as clinical status of the child was reported to the study nurse based at the nearest RHF, recorded into the study database and a home visit scheduled for 28 days since provisional enrolment.

#### During admission (RHF)

The high percentage of children (67%) that successfully completed referral to a designated RHF was assessed and treated according to national guidelines [35]. Trained CARAMAL study nurses extracted key patient information such as signs at symptoms on arrival, test results, diagnosis, treatment provided, daily clinical assessments, and condition of the child at discharge from facility records.

#### Follow-up home visits

Home visits consisted of face-to-face interview with parent/guardian and child’s blood testing 28 to 30 days after provisional enrolment. Finger or heel-prick capillary blood was collected from all children for (1) malaria antigen testing (CareStartTM malaria HRP2 or HRP2/pLDH combined mRDT, Access Bio, Ethiopia), and (2) haemoglobin (Hb) level measurement (HemoCue Hb 201, Ängelholm, Sweden). Interviews focused on the child’s current health status and retrospectively recorded the history of fever, signs and symptoms, including RAS, the treatment-seeking pathway during the past 28 days and treatment(s) received. For deceased children, the circumstances and possible causes of death were elicited 4–8 weeks after their passing, to respect the mourning period.

*Data collection tools* We used structured electronic data collection forms designed on the Open Data Kit platform (ODK, https://opendatakit.org/) to capture data at each point of contact: at day 0, during admission in a RHF, and during the day-28 home visit. Each enrolled child was assigned a unique CARAMAL identification number in order to link the data collected at different points.

### Study outcomes

The primary outcome of this study was the child’s health status on day 28 home visit as reported by his (her) parent/guardian: (healthy, still sick or deceased). Secondary outcomes consisted of three binary variable defining key elements of the case management process: (1) RAS administration (yes/no); (2) referral completion to a dedicated RHF (yes/no); (3) provision of an injectable anti-malarial treatment at the RHF (yes/no). Exposure variables of interest were the presence of the danger signs listed above, defined as a categorical variable, and including both “iCCM general danger signs” and “DRC-specific iCCM danger signs. In addition, covariates of interest included enrolment location (CHW/PHC), Health Zone (Ipamu, Kenge and Kingandu), malaria test result at the RHF (positive/negative or not done), severe anaemia (Hb < 5 g/dL versus Hb ≥ 5 g/dL), blood transfusion (yes/no), malaria oral treatment after parenteral treatment (yes/no), malaria test result on day 28 (positive/negative or not done), and anaemia (Hb < 11 g/dL versus Hb ≥ 11 g/dL) on day 28.

### Sample size calculation and statistical analysis

The overall sample size of the CARAMAL multi-country study was estimated for the primary outcome (mortality at Day 28) across the three project countries. The CFR was assumed to be 6% at baseline (historical CFR for severe malaria: 2.8% MATIAS Study DRC [12], 8.5% AQUAMAT [8]). Over the three countries, a minimum of 6,032 severe malaria cases in children < 5 years were required over 24 months to detect a 30% reduction in CFR between a 6 months baseline and 18 months following the roll-out of RAS, with 80% power and α = 0.05, as described in [33]. This was a very large sample size that was amply sufficient for the analysis presented here.

Given the large sample size required for measuring the impact of RAS on CFR in each country, the sample size for the secondary analysis presented here was largely sufficient [32].

Data were analysed in STATA version 16.0 (STATA Corporation, College Station, TX, USA). An Intention-to-Treat (ITT) analysis was done, which included all participants who were formally enrolled following informed consent, and for whom day-28 follow-up data were available. The distribution of danger signs and symptoms among participants was computed, stratified in study phases (pre-RAS and post-RAS periods), as well as by RAS users and RAS non-users. Continuous variables were summarized by their mean and standard deviation (SD), or median and interquartile range (IQR) when the distribution was skewed. Dichotomous outcomes were summarized as proportions, with 95% confidence intervals (95%CI). We used the Pearson Chi square test to compare proportions. Finally, we built a multilevel-mixed effects logistic regression models for each primary and secondary outcome to adjust for potential confounders and included enrolling provider as random effect to adjust for clustering at that level. Results are presented as adjusted odd ratios (aOR) with their 95%CI.

### Ethics

The CARAMAL study protocol was approved by the Research Ethics Review Committee of the World Health Organization (WHO ERC, No. ERC.0003008), the Ethics Committee of the University of Kinshasa School of Public Health (No. 012/2018), and the Scientific and Ethical Review Committee of CHAI (No. 112, 21 Nov 2017). The study was registered on ClinicalTrials.gov (NCT03568344). Consent was obtained provisionally from parent/guardians of the sick child prior at first point of contact. Given the urgency of the child’s condition, it was not deemed adequate to perform a full informed consent that that point. This was then done once the child reached the RHF.

## Results

### Characteristics of study participants

The study flow-chart (Additional file 3: Fig. S3) displays recruited study participants and their subsequent case management until their day-28 outcome assessment.

Key characteristics of study participants are shown in Table 1. Between June 2018 and July 2020, a total of 3042 febrile children < 5 years old (median age 2 years [IQR 1–3]) seeking care from a CHW or PHC provider were recruited into the study. Of those, 57.6% were children aged 0–2 years and 46.9% were female, with no difference in sex ratio between the pre-RAS and post-RAS periods (p = 0.93). Overall, in Kingandu HZ, significantly less children were recruited (813) compared to Kenge (1101) and Ipamu (1128) HZs. The vast majority of participants were enrolled at the PHC level (94.6%) rather than by CHWs (5.4%). Overall, 67% of patients successfully completed referral to a dedicated RHF, and 1/3 (33.5%) were anaemic upon arrival at the RHF, without change between the pre-RAS and post-RAS periods.

**Table 1:** Characteristics of study participants at enrolment, by study phase

Variable	Overall N = 3042	Pre-RAS N = 761	Post-RAS N = 2281	P-value comparing pre-post RAS
	%	%	%	
Age				0.80
0–2 years	57.6	57.2	57.7	
2–5 years	42.4	42.8	42.3	
Sex				0.93
Male	53.1	53.2	53.1	
Female	46.9	46.8	47.0	
Health Zone				< 0.001
Ipamu	37.1	30.1	39.4	
Kenge	36.2	40.9	34.6	
Kingandu	26.7	29.0	26.0	
Enrolment location				< 0.001
CHW	5.4	7.9	4.6	
PHC	94.6	92.1	95.4	
General iCCM danger signs				< 0.001
No	33.6	46.7	29.2	
Yes	66.4	53.4	70.8	
Referral completion				0.81
No	33.6	33.1	33.8	
Yes	66.4	66.9	66.2	
Malaria test				0.002
Negative/Not done	47.8	52.7	46.2	
Positive	52.2	47.3	53.8	
Anaemia				0.06
No/mild anaemia/not determined	66.5	69.3	65.6	
Severe anaemia (≤ 5 g/dL)	33.5	30.7	34.4	

CHW: Community Health Worker; PHC: Primary Health Care; RHF: Referral Health Facility; RAS1: rectal artesunate; iCCM: integrated Community Case Management

Nearly two-thirds of patients (66.4%) presented iCCM general danger signs upon enrolment (Table 1). This proportion rose markedly from 53.4% (pre-RAS) to 70.8% (post-RAS), p < 0.001. Table 2 shows that “Convulsion” was the most frequent danger sign reported (40.8%), followed by “Not able to breastfeed, drink or eat anything” (36.2%) and “unusually sleepy or unconscious” (18.9%) with a significantly higher proportion of children presented during post-RAS compared to pre-RAS study phase (p < 0.001). Among DRC-specific iCCM danger signs, “unable to sit or stand up” was most frequently reported (26.1%), with a higher proportion during post-RAS phase (p < 0.001).

**Table 2:** Danger signs triggering RAS among children < 5 years recruited at community level, by study phase

Variable	Overall N = 3042	Pre-RAS N = 761	Post-RAS N = 2281	P-value comparing pre-post RAS
	%	%	%	
iCCM general danger signs				
Convulsions	40.8	30.4	44.2	< 0.001
Not able to breastfeed, drink or eat anything	36.2	31.3	37.8	0.001
Unusually sleepy or unconscious	18.9	23.7	17.3	< 0.001
Vomiting everything	8.5	8.9	8.3	0.58
DRC-specific iCCM danger signs				
Unable to sit or stand up	26.1	9.7	31.6	< 0.001
Weakness or asthenia	17.4	16.4	17.7	0.43

iCCM: integrated Community Case Management; RAS: rectal artesunate

The results that follow include the use of RAS, and are therefore restricted to 2281 patients enrolled during the post-RAS phase (April 2019 to July 2020) of the study. Tables 3, 4, 5, 6 and 7 show how key co-variates as well as the reported danger signs are associated with a number of operational and health outcomes.

**Table 3:** Determinants of RAS use by peripheral health workers

Determinant	N	%	Adjusted OR	95% CI	*p*-value
Age					
0–2 years	1316	57.7	Ref.		
2–5 years	965	42.3	1.58	1.20–2.08	0.001
Sex					
Male	1210	53.0	Ref.		
Female	1071	47.0	1.02	0.79–1.31	0.90
Enrolment location					
CHW	104	4.6	Ref.		
PHC	2177	95.4	0.87	0.40–1.89	0.72
Health Zone					
Ipamu	899	39.4	Ref.		
Kenge	790	34.6	0.69	0.41–1.18	0.17
Kingandu	592	26.0	0.48	0.28–0.84	0.01
Danger signs					
No/Others	415	18.2	Ref.		
Yes (iCCM general danger signs)	1614	70.8	2.77	2.04–3.77	< 0.001
Weakness or asthenia	103	4.5	1.19	0.64–2.19	0.58
Unable to sit	149	6.5	2.06	1.12–3.80	0.02

N = 2281. OR: Odds ratio; CHW: Community Health Worker; PHC: Primary Health Care; 95% CI: 95% confidence interval

**Table 4:** Estimated associations between selected determinants and referral completion

Determinant	N	%	Adjusted OR	95% CI	p-value
Age					
0–2 years	1316	57.7	Ref.		
2–5 years	965	42.3	0.71	0.54–0.93	0.013
Enrolment location					
CHW	104	4.6	Ref.		
PHC	2177	95.4	4.22	1.09–16.32	0.037
Health Zone					
Ipamu	899	39.4	Ref.		
Kenge	790	34.6	0.10	0.03–0.29	< 0.001
Kingandu	592	26.0	0.50	0.17–1.50	0.22
Danger signs					
No/Others	415	18.2	Ref.		
Yes (iCCM general danger signs)	1614	70.8	1.01	0.72–1.43	0.95
Weakness or asthenia	103	4.5	1.35	0.64–2.86	0.44
Unable to sit	149	6.5	1.89	1.01–3.54	0.08
RAS administration					
Yes	1954	85.7	Ref.		
No	327	14.3	0.63	0.44–0.92	0.02
Mean of transport					
Going by foot	1910	83.7	Ref.		
Other mean	371	16.3	0.89	0.61–1.30	0.56

N = 2281. OR: Odds ratio; CHW: Community Health Worker; PHC: Primary Health Care; 95% CI: 95% confidence interval; RAS: rectal artesunate; Ref.: Reference

**Table 5:** Determinants of injectable antimalarial treatment for severe malaria at referral health facilities in community enrolments

Determinants	N	%	Adjusted OR	95% CI	p value
Age					
Children (0–2 years)	921	61.0	Ref.		
Children (2–5 years)	590	39.0	1.13	0.78–1.63	0.53
Enrolment location					
CHW	40	2.7	Ref.		
PHC	1471	97.4	0.57	0.17–1.91	0.36
Health Zone					
Ipamu	716	47.4	Ref.		
Kenge	500	33.1	6.30	3.30–12.05	< 0.001
Kingandu	295	19.5	0.83	0.48–1.44	0.51
Danger signs					
No/Others	271	17.9	Ref.		
Yes (iCCM general danger signs)	1049	69.4	1.12	0.70–1.78	0.64
Weakness or asthenia	68	4.5	1.16	0.45–2.98	0.76
Unable to sit	123	8.1	1.39	0.61–3.13	0.43
RAS administration					
No	220	14.6	Ref.		
Yes	1291	85.4	4.75	3.00–7.52	< 0.001
Referral delay					
0–1 day	1066	70.6	Ref.		
> 1 day/Not documented	445	29.4	1.05	0.71–1.55	0.81
Malaria test result (RHF)					
Positive	1227	81.2	Ref.		
Negative/Not done	284	18.8	0.07	0.04–0.11	< 0.001
Anaemia at arrival at RHF					
No/mild anaemia/not done	726	48.1	Ref.		
Severe anaemia (≤ 5 g/dL)	785	52.0	2.28	1.38–3.77	0.001
Other comorbidities					
No	802	53.1	Ref.		
Yes	709	46.9	2.36	1.62–3.44	< 0.001
Blood transfusion					
Yes	775	51.29	Ref.		
No	736	48.71	0.53	0.32–0.87	0.01

N = 1511. OR: Odds ratio; CHW: Community Health Worker; PHC: Primary Health Care; RHF: Referral Health Facilities; RAS: rectal artesunate; 95% CI: 95% confidence intervals

**Table 6:** Estimated associations between selected factors and the health status of febrile children 28 days after initial contact with the health system (cured versus still sick)

Determinants	N	%	Adjusted OR	95% CI	*p-*value
Age					
Children (0–2 years)	1198	56.5	Ref.		
Children (2–5 years)	922	43.5	0.83	0.63–1.10	0.20
Health Zone					
Ipamu	842	39.7	Ref.		
Kenge	734	34.6	1.48	1.05–2.07	0.02
Kingandu	544	25.7	0.62	0.40–0.97	0.04
Danger signs					
No/Others	392	18.5	Ref.		
Yes (iCCM general danger signs)	1477	69.7	1.08	0.75–1.55	0.68
Weakness or asthenia	103	4.9	1.16	0.59–2.28	0.67
Unable to sit	148	7.0	1.13	0.61–2.12	0.70
RAS administration					
No	306	14.4	Ref.		
Yes	1814	85.6	0.64	0.45–0.92	0.02
Injectable antimalarial					
No/NA	928	43.8	Ref.		
Yes	1192	56.2	1.03	0.67–1.59	0.89
Oral antimalarial given at RHF					
No	996	47.0	Ref.		
Yes	1124	53.0	1.08	0.68–1.72	0.74
Oral treatment given at discharge or prescribed					
No	1432	67.6	Ref.		
Yes	688	32.5	1.12	0.76–1.64	0.58
Malaria test result on day 28					
Negative/not done	1279	60.3	Ref.		
Positive	841	39.7	4.67	3.47–6.30	< 0.001
Anaemia (day 28)					
No anaemia/not done	790	37.3	Ref.		
Anaemia (Hb < 11 g/dL)	1330	62.7	2.01	1.46–2.77	< 0.001

N = 2120 alive on Day 28. OR: odds ratio; CHW: Community Health Worker; PHC: Primary Health Care; RHF: Referral Health Facilities; RAS: rectal artesunate; ACT: artemisinin-based combination therapy; 95% CI: 95% confidence interval; Hb: Haemoglobin; NA: not applicable (because not at RHF)

**Table 7:** Determinants of death within 28 days following enrolment

Determinants	N	%	Adjusted OR	95% CI	*p* value
Age					
Children (0–2 years)	1255	57.6	Ref.		
Children (2–5 years)	923	42.4	0.44	0.29–0.65	< 0.001
Health Zone					
Ipamu	845	38.8	Ref.		
Kenge	749	34.4	0.66	0.35–1.24	0.19
Kingandu	584	26.8	0.78	0.41–1.50	0.45
iCCM danger signs					
No/Others	415	19.1	Ref.		
Yes (iCCM general danger signs)	1614	74.1	1.57	0.94–2.61	0.08
Unable to sit	149	6.8	0.14	0.02–1.13	0.07
RAS administration					
No	308	14.1	Ref.		
Yes	1870	85.9	1.50	0.86–2.60	0.15
Malaria test (RHF)					
Negative/Not done	999	45.9	Ref.		
Positive	1179	54.1	1.89	0.98–3.65	0.06
Anaemia on arrival at RHF					
No/mild anaemia/not done	1430	65.7	Ref.		
Anaemia (Hb < 5 g/dL)	748	34.3	2.13	1.22–3.69	0.008
Other comorbidities					
No	1501	68.9	Ref.		
Yes	677	31.1	1.13	0.67–1.91	0.64
Injectable antimalarial					
No/NA	970	44.5	Ref.		
Yes	1208	55.5	2.07	0.72–5.95	0.18
Oral antimalarial given at RHF					
No	1076	49.4	Ref.		
Yes	1102	50.6	0.13	0.07–0.26	< 0.001
Oral treatment given at discharge or prescribed					
No/NA	1499	68.8	Ref.		
Yes	679	31.2	0.53	0.25–1.13	0.10
Injectable antimalarial & ACT					
No	920	42.2	Ref.		
Yes	1258	57.8	0.26	0.09–0.79	0.018

N = 2178. OR: Odds ratio; CHW: Community Health Worker; iCCM: integrated Community Case Management; PHC: Primary Health Care; RHF: Referral Health Facility; RAS: rectal artesunate; 95% CI: 95% confidence intervals; ACT: artemisinin-based combination therapy; Hb: Haemoglobin; NA: not applicable

### Outcome 1: RAS use

The contribution of different predictors associated with RAS use at CHW and PHC level is shown in Table 3. Sick children aged 2–5 years were more likely to receive RAS compared to those aged 0–2 years (aOR = 1.58, 95% CI 1.20–2.08). There was no evidence of significant association between RAS use and gender or enrolment location. Significant heterogeneity in RAS use was observed among the three HZ.). Children with one of the iCCM general danger signs were significantly more likely to receive RAS (aOR = 2.77, 95% CI 2.04–3.77), suggesting a good recognition of these signs at primary care level. The same was true for those “unable to sit” (aOR = 2.06, 95% CI 1.12–3.80), but not for children suffering from weakness or asthenia (aOR = 1.19, 95% CI 0.64–2.19).

### Outcome 2: Referral completion

Predictors associated with referral completion are presented in Table 4. Children in the age group of 2 to 5 years were significantly less likely to complete referral to a RHF (aOR = 0.71, 95% CI 0.54–0.93) than younger children. Compared to children enrolled by a CHW, PHC enrolments were associated with much higher odds of completing referral (aOR = 4.22, 95% CI 1.09–16.32). Since these results are controlled for signs of severity, there is clearly a differentiated recommendation between both settings. Clearly, referral completion rates appeared lower in Kenge and Kingandu compared to Ipamu HZ, but a statistically significant decrease was only observed for Kenge HZ (aOR = 0.10, 95% CI 0.03–0.29). This surprised us because Ipamu is the most remote location. Referral completion seemed only to be related to the identified “unable to sit” (aOR = 1.89, 95% CI 1.01–3.54) but not any of the other danger signs, which seem to trigger the same referral patterns. Importantly, patients who did not receive RAS were significantly less likely to complete referral (aOR = 0.63, 95% CI 0.44–0.92). Finally, using other means of transport including bicycle, motorbike and car did not show a significant association with referral completion compared to those reaching the RHF by foot.

### Outcome 3: Injectable treatment provision at RHF

For the injectable treatment provision outcome, we assessed determinants for the 1511 children that completed referral successfully, and were thus eligible for injectable treatment (artesunate, artemether or quinine) while admitted (Table 5). There was no evidence of association between the provision of an injectable anti-malarial and age of children or enrolment location (CHW or PHC). Injectable treatment was significantly more likely to be administered in Kenge (aOR = 6.30, 95% CI 3.30–12.05). At this point of the case management process, none of the danger signs recognized at primary level seemed to be associated with injectable treatment, which was expected. On the other hand, patients treated with RAS were much more likely to receive injectable treatment (aOR = 4.75, 95% CI 3.00–7.52) and that was unexpected. Timing of referral was not significantly associated with increased odds of injectable anti-malarial treatment provision And logically, patients tested negative for malaria or who did not have tested had much lower odds of injectable treatment provision, aOR = 0.07, 95% CI 0.04–0.11.Severe anaemia and receiving a blood transfusion were associated with a higher injectable frequency.

### Outcome 4: Determinants of health status on day 28 (well versus still sick, among survivors)

For this outcome, we only included children recruited during post-RAS phase of the study that still alive during home visits. Table 6 displays the odds to be cured versus still sick among the 2120 children still alive on Day 28 home visits, of which 1846 (87.1%) were healthy and 274 (12.9%) were sick. Nearly 40% of the children still had a positive mRDT on Day 28 (39.7%). It appears that age did not show evidence of association with the health status on day 28. The odds of still being sick were higher in Kenge (aOR = 1.48, 95% CI 1.05–2.07) compared to Ipamu (Ref) and lower in Kingandu (aOR = 0.62, 95% CI 0.40–0.97) compared to Ipamu. None of the initial danger signs were predictive of clinical cure on Day 28. Importantly, patients who received RAS were less likely to be sick on day 28 (aOR = 0.64, 95% CI 0.45–0.92) compared to those who did not. On the other hand, RHF treatment did not seem to make a difference to Day 28 health status in this group of children. Counter-intuitively, patients with a positive test for malaria on day 28 or with at least mild anaemia were significantly more likely to still sick at that time point (aOR = 4.67, 95% CI 3.47–6.30 and aOR = 2.01, 95% CI 1.46–2.77).

### Outcome 5: Death within 28 days after enrolment

For the case fatality ratio calculation, all 2281 children enrolled into the PSS during the post-RAS phase were included in the denominator. However, while assessing determinants of deaths the same sample after exclusion of 103 children that presented “weakness or asthenia”, which was a danger sign that did not contribute to this outcome (death) was analysed. By the time of the Day 28 visit, a total of 161 participants were deceased among the 2281 children in the post-RAs phase (CFR: 161/2,281 = 7.1% (95% CI 6.1–8.2)). The great majority (137 or 85.1%) displayed iCCM general danger signs at enrolment and 24 showed other or DRC-specific iCCM danger signs (Additional file 4: Table S1). Because “weakness or asthenia” (N = 103) was shown not to be a predictor of death, these 103 children were therefore excluded, resulting in 2178 children of whom determinants of death within 28 days following enrolment were analyzed (Table 7). Compared to children between 0 and 2 years old, children of age 2 to 5 years were less likely to die (aOR = 0.44, 95% CI 0.29–0.65). The odds of dying were higher but not significantly different between children presenting iCCM general danger signs compared to those that did not show these signs (aOR = 1.57, 95% CI 0.94–2.61), while they were lower but not significantly among children “unable to sit” (aOR = 0.14, 95% CI 0.02–1.13).

The odds of dying were 1.50 times higher in patients that did receive RAS but the difference was not significant, since the confidence interval was rather large (95% CI 0.86–2.60); nevertheless this is an encouraging finding for RAS administration. Clearly, patients with either a positive malaria test at the RHF (aOR = 1.89, 95% CI 0.97–3.62) and especially with severe anaemia (aOR = 2.13, 95% CI 1.22–3.69), had increased odds of dying.

Injectable treatment given alone did not influence mortality. By contrast, the provision of an oral ACT at the RHF, either given directly or as a prescription, did offer significant protection. The full course of treatment as recommended in the national guidelines offered a high protection against dying (aOR = 0.26, 95% CI 0.09–0.79) this obviously points towards the importance of proper case management of severe malaria cases.

## Discussion

In the CARAMAL study, the recognition of danger signs and symptoms of severe febrile illness by community-based providers (CHW and PHC) was the starting point for enrolling a child. Firstly, this allowed to assess and classify sick children according to the iCCM or IMCI algorithms [15, 16]. Secondly, it allowed initiating the proper course of action for the child, including early treatment and particularly the administration of RAS followed by a recommendation for referral to a higher-level facility. While the evaluation of the overall effectiveness of RAS is the topic of another publication [33], we here investigated the value of danger signs and other factors as predictors for appropriate case management and health outcomes, including mortality.

As with any observational study designs, this study had some methodological limitations. The analysis presented here focused on an individual patient analysis, for which many indicators were collected. To some extent, relevant confounders could be controlled for in the multivariate analysis, but it was impossible to avoid residual confounding, especially from the many health system factors that are presented below. Data on socio-economic status would certainly have been important to include in this study analysis but the decision taken was to focus on care seeking in the Day-28 interview, which could not be extended indefinitely. A second major limitation was that despite the intensity of the field work, it was impossible to track the clinical condition of the children continuously for 28 full days. The field staff did their best to re-construct the treatment-seeking pathway during the Day 28 interview, focusing on issues such as location of care, treatment received, and referrals, but there was certainly a risk of recall bias, despite major efforts through training and supervision. These results were then consolidated with the observations from our study nurses at the RHF, if the children were brought there. This still left some large gaps because the use of multiple providers, public and private, was the norm rather than the exception [36].

In DRC, two danger signs used by health care workers were not part of the traditional iCCM general danger signs. Findings from this study suggest that the most frequently reported alternative danger sign was “unable to sit or stand up” (26.1%), which is similar to “unusually sleepy or unconscious” among the iCCM general danger signs. Of note, the relative frequency of iCCM general danger signs appeared to increase during the post-RAS phase compared to the pre-RAS phase. This could be the results of community sensitization and training of health workers prior to RAS rollout. Unfortunately, there was no independent measure to confirm this.

Little is known from the scientific literature about the frequency and importance of danger signs and how they predict RAS provision, referral, subsequent case management at a RHF, and ultimately the child’s health outcome. These are some important findings by the CARAMAL project. In an earlier multi-country cluster randomized controlled trial conducted in Ghana, Guinea-Bissau, Tanzania and Uganda using pre-referral RAS at community level [37], the odds ratio of being treated with RAS when a child presented danger signs was 1.84 (95% CI 1.20–2.83); p = 0.005. These findings are consistent with our results showing that those who presented iCCM general danger signs were significantly more likely to receive RAS (aOR = 2.77, 95% CI 2.04–3.77). The trend was the same for the two additional DRC-specific signs triggering RAS use, although the association was not significant in children suffering from “weakness or asthenia”. Findings from Liberia have shown that the proportions of correct diagnosis and treatment by community-based healthcare providers varied substantially for uncomplicated disease, but consistency was better for more severe cases, even though the accurate recognition of danger signs was sub-optimal [38]. Findings from this study suggest that danger signs increase substantially the probability of receiving RAS, but not subsequent referral and treatment at a RHF. This clearly point towards the fact that the health care workers at primary level follow better the treatment guidelines than their peers in RHF.

Other reasons for the proper recognition of signs of severity and appropriate administration of RAS were observed between the three study Health Zones, due to differences in the availability of RAS (more or less stock-outs), leadership issues of local health authorities (at both HZ and PHC level), coverage in CHW and PHC within each HZ, and finally also health workers’ knowledge and skills. Throughout the study implementation period, Kingandu HZ had consistently better stock of essential commodities including RAS, injectable drugs and ACT. It experienced fewer changes in leadership compared to the other two HZ, and this might be a reason for such good operational results. These results point towards the fact that complex care interventions such as the management of a severely ill child requires many health system factors to align to be successful. It also highlights the importance of doing such “real-world” intervention studies to document with some rigor these issues. However, it is unfortunately also clear than many of these operational factors cannot be fully accounted for in a quantitative analysis because they are too many and often difficult to measure and/or quantify (such for example as the quality of leadership). Conversely, one small study in Zambia showed that when all the health system factors align properly, including transports for referrals, then CFR from malaria and other severe causes decreases massively [39].

One of the main purposes of RAS is to allow a safer referral, since lower level health facilities and CHW are not supposed to use injectable anti-malarials. Hence, CARAMAL investigated referral determinants in detail. In contrast to result found in Uganda [40], in DRC young patients (0–2 years), patients recruited at PHCs as well as those treated with RAS, were significantly more likely to complete referral. The fact that infants are at a higher risk of complications and especially death would almost certainly explain why younger children had higher referral rates. Similarly to the results from another study in DRC [41], this study found that RAS administration was significantly associated with increased odds of completing referral. This contrasts with findings from a study in Uganda in which nearly all children treated with pre-referral RAS failed to comply their referral [42]. Possibly, this may be explained in this study by the intensive sensitization of caretakers and health workers during training prior to RAS rollout. Surprisingly, no evidence was found for an association between referral completion and presence of iCCM general danger signs. This does not match evidence from another study in Uganda [40]. Additional factors based on our anecdotal experience and reported in the literature are logistics, finances of the patients, communication skills, perceived quality of care, lack of time and need to care for other children and an improvement in the child’s condition [40, 42–44]. This is another example of the complexity of the decision-making process for this health-seeking step that involves a substantial time and money investment.

Findings from this study suggest that injectable treatment alone did not seem to significantly decrease the odds of dying. This is an important finding, which was also documented in the two other CARAMAL countries (Nigeria and Uganda, results not shown). RAS followed by a few doses of an injectable anti-malarial (mostly artesunate) constitutes an incomplete monotherapy treatment. Hence, it is not surprising that this makes little difference to the outcome of the child.

By contrast, oral anti-malarial treatment including an ACT or oral quinine while admitted in a RHF was very significantly associated with a large decrease in the odds of dying (by 87%). The same strong effect (a 74% reduction in the risk of dying) was seen for the combination of parenteral malaria treatment followed by an ACT, as recommended by the WHO treatment guidelines [6]. Again, this is consistent with findings in the other two CARAMAL countries as reviewed by Signorell et al. [45]. This importance of the oral anti-malarial treatment following injectable treatment in RHF is a very important finding from our study for three reasons: (1) its favorable effect on the health outcomes of these children, (2) because of the threat posed by artesunate monotherapy for the development of drug resistance (Awor et al. pers. commun.), and (3) because it is an actionable issue since artemisinin-based combinations are widely available in endemic countries. Finally, the odds of dying were 1.50 times higher in patients who did not received RAS, although the difference was on the margins of statistical significance. DRC key findings are consistent with findings in Nigeria and Uganda, and point towards the fact that RAS can work in reducing mortality, but it does not work well as a single intervention. RAS can only become effective in the frame of a functioning health systems that includes a functioning referral system, and especially an improved quality of case management in RHFs. In contrast to previous RCTs [9] demonstrating the health benefits of RAS pre-referral administration, this study demonstrates the real-world limitations of this intervention, and hence carries an important and actionable message for health authorities and the global health community.

## Conclusion

This study aimed at describing key elements of case management for suspected severe cases of malaria, as well as the distribution of signs and symptoms among children < 5 years. The differences in case management of children < 5 years with different danger signs and varying treatment pathways, and related these to referral patterns, treatment at RHF, and key health outcomes including mortality were investigated. This study’s main findings point towards the fact that danger signs are reasonably well-recognized by health provider at the primary care level, and that RAS could influence positively health outcomes of such severe disease episodes. Its effectiveness is clearly hampered by the insufficient quality of care at RHF, especially the provision of a full course of an ACT following parenteral treatment. These are simple but important findings, that requires urgent action by the health system planners and implementers, and which have a great potential to improve child survival in highly endemic malaria settings. 

## Supplementary Information

**Additional file 1: Figure S1. **Map displaying the three CARAMAL study health zones in the Democratic Republicof the Congo.

**Additional file 2: Figure S2. ** iCCM general danger signs and DRC-specific iCCM danger signs.

**Additional file 3: Figure S3. **Inclusion flow-chart.

**Additional file 4: Table S1. **Number of deaths among community enrolment during post-RAS per iCCM general danger signs and DRC-specific iCCM danger signs. N = 2281.

## Data Availability

The datasets used and/or analyzed during the current study are available from the corresponding author upon reasonable request.

## Abbreviations

95% CI: 95% Confidence intervals
ACT: Artemisinin-based combination therapy
aOR: Adjusted odds ratio
CARAMAL: Community access to rectal artesunate for malaria
CFR: Case fatality ratio
CHCS: Community Health Care Site
CHW: Community Health Worker
DHS-DRC II: DRC second Demographic and Health Survey
DRC: Democratic Republic of the Congo
g/dL: Gram per deciliter
Hb: Haemoglobin
*HRP2*: *Plasmodium falciparum* antigen Histidine Rich Protein 2
HZ: Health Zone
iCCM: Integrated community case management
IMCI: Integrated Management of Childhood Illness
IQR: Interquartile range
ITT: Intention-to-Treat
mRDT: Malaria Rapid diagnostic test
NA: Not applicable
ODK: Open Data Kit platform
OR: Odds ratio
PHC: Primary Health Care facilities
pLDH: Plasmodium lactate dehydrogenase
PSS: Patient Surveillance System
RAS: Rectal artesunate
RHF: Referral Health Facility
SM: Severe malaria
SD: Standard deviation
Swiss TPH: Swiss Tropical and Public Health Institute
UNICEF: The United Nations Children's Fund
WHO: The World Health Organization
