# Supplementary material for: Key factors predicting suspected severe malaria case management and health outcomes: an operational study in the Democratic Republic of the Congo
Source: Malar J. 2022 Sep 27;21:274. doi: 10.1186/s12936-022-04296-2 (PMC9513903; doi:10.1186/s12936-022-04296-2)

Supplementary Figure S1: **Map displaying the three CARAMAL study health zones in the Democratic Republic of the Congo.**


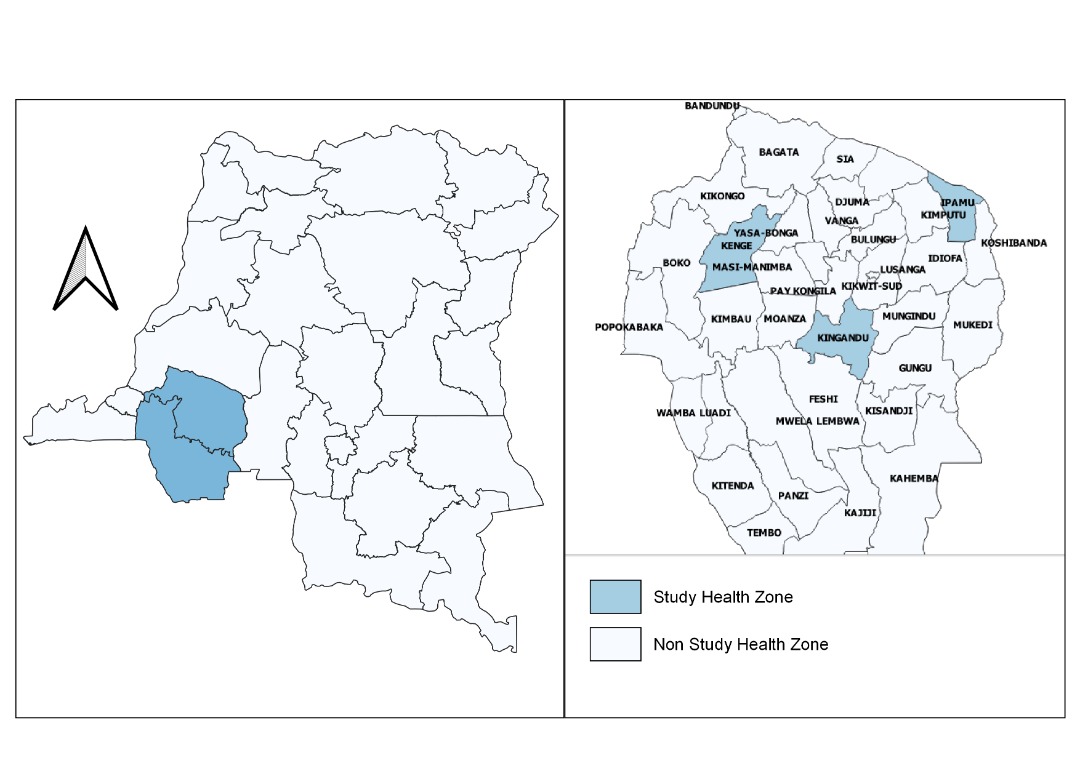

Supplement: Supplementary file 1 — Additional file 1: Figure S1. Map displaying the three CARAMAL study health zones in the Democratic Republicof the Congo. [file 12936_2022_4296_MOESM1_ESM.docx]
