# Supplementary material for: Key factors predicting suspected severe malaria case management and health outcomes: an operational study in the Democratic Republic of the Congo
Source: Malar J. 2022 Sep 27;21:274. doi: 10.1186/s12936-022-04296-2 (PMC9513903; doi:10.1186/s12936-022-04296-2)
Supplement: Supplementary file 2 — Additional file 2: Figure S2. iCCM general danger signs and DRC-specific iCCM danger signs. [file 12936_2022_4296_MOESM2_ESM.docx]

Supplementary Figure S2: **iCCM general danger signs and DRC-specific iCCM danger signs**

- **“*iCCM general danger signs*”:** This concept consists of the general danger signs according to the iCCM algorithm including (1) vomiting everything, (2) convulsions, (3) not being able to drink/eat, and (4) being very sleepy or even unconscious (12). The presence of at least one of these danger signs triggers RAS administration and immediate referral in children under 6 years old at community level (2, 14).
- ***“DRC-specific iCCM danger signs”:*** These danger signs refer to other two additional signs/symptoms in DRC identifying a child as eligible for referral and hence RAS pre-referral treatment: (1) being “unable to sit or stand up” and (2) “weakness or asthenia”.

Vomiting everything

Convulsions

Not being able to drink/eat

Being very sleepy or even unconscious

Being unable to sit or stand up

Weakness or asthenia

**DRC-specific iCCM danger signs**

**iCCM general danger signs**

**Danger signs**
