# Supplementary material for: Key factors predicting suspected severe malaria case management and health outcomes: an operational study in the Democratic Republic of the Congo
Source: Malar J. 2022 Sep 27;21:274. doi: 10.1186/s12936-022-04296-2 (PMC9513903; doi:10.1186/s12936-022-04296-2)

Supplementary Figure S3: **Inclusion flow-chart**.

**ITT = Intension-to-Treat. RHF = Referral Health Facility. iCCM = integrated Community Case Management. RAS = Rectal Artesunate. Ref. = Referral completed. Inject. = Injectable antimalarial treatment.**


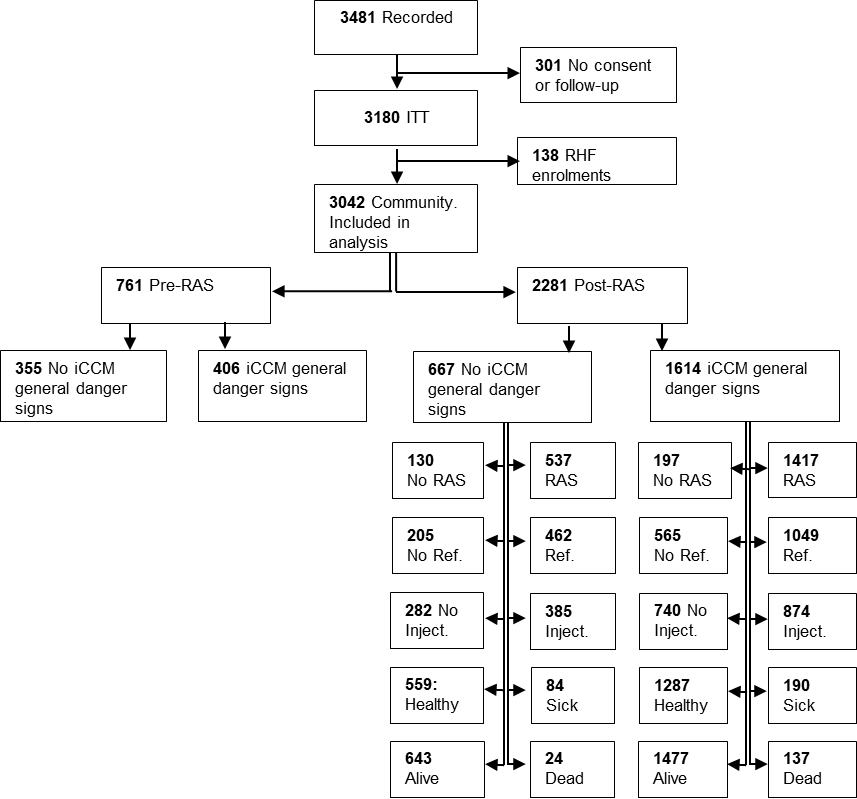

Supplement: Supplementary file 3 — Additional file 3: Figure S3. Inclusion flow-chart. [file 12936_2022_4296_MOESM3_ESM.docx]
