# Supplementary material for: Key factors predicting suspected severe malaria case management and health outcomes: an operational study in the Democratic Republic of the Congo
Source: Malar J. 2022 Sep 27;21:274. doi: 10.1186/s12936-022-04296-2 (PMC9513903; doi:10.1186/s12936-022-04296-2)
Supplement: Supplementary file 4 — Additional file 4: Table S1. Number of deaths among community enrolment during post-RAS per iCCM general danger signs and DRC-specific iCCM danger signs. N = 2281. [file 12936_2022_4296_MOESM4_ESM.docx]

Supplementary Table S1: **Number of deaths among community enrolment during post-RAS per iCCM general danger signs and DRC-specific iCCM danger signs. N = 2281**

| **Dead** | **iCCM general danger signs** | |  | **DRC-specific iCCM danger signs** | | **Total** |
| --- | --- | --- | --- | --- | --- | --- |
|  | Yes | No/Others |  | Not able to sit | Weakness or asthenia |  |
|  |  |  |  |  |  |  |
| No | 1477 | 392 |  | 148 | 103 | **2120** |
| Yes | 137 | 23 |  | 1 | 0 | **161** |
|  |  |  |  |  |  |  |
| **Total** | **1614** | **415** |  | **149** | **103** | **2281** |
|  |  |  |  |  |  |  |
